# Distinct phenotypes of platelet, monocyte, and neutrophil activation occur during the acute and convalescent phase of COVID-19

**DOI:** 10.1080/09537104.2021.1921721

**Published:** 2021-05-17

**Authors:** Yashuan Chao, Johan Rebetz, Anna Bläckberg, Gisela Hovold, Torgny Sunnerhagen, Magnus Rasmussen, John W. Semple, Oonagh Shannon

**Affiliations:** 1 Division of Infection Medicine, Department of Clinical Sciences, Lund, Faculty of Medicine, Lund University, Lund, Sweden; 2 Division of Hematology and Transfusion Medicine, Faculty of Medicine, Lund University, Sweden; Clinical Immunology and Transfusion Medicine, Office of Medical Services, Region Skåne, Lund, Sweden; 3 Skåne University Hospital, Lund, Sweden; 4 Clinical Microbiology, Office of Medical Services, Region Skåne, Lund, Sweden

**Keywords:** Blood platelets, COVID-19, monocytes, neutrophils, platelet activation

## Abstract

SARS-CoV-2 has spread rapidly worldwide, causing the COVID-19 pandemic. Platelet activation and platelet-leukocyte complex formation are proposed to contribute to disease progression. Here, we report platelet and leukocyte activation during acute and convalescent COVID-19 in patients recruited between May-July 2020. Blood samples were analyzed by flow cytometry and ELISA using paired comparison between inclusion (day 0) and 28 days later. The majority of patients were mildly or moderately ill with significantly higher cytokine levels (IL-6 and IL-10) on day 0 as compared with day 28. Platelet activation and granule release were significantly higher on day 0 compared with day 28, as determined by ADP- or thrombin-induced surface CD62P expression, baseline released CD62P, and thrombin-induced platelet-monocyte complex formation. Monocyte activation and procoagulant status at baseline and post activation were heterogeneous but generally lower on day 0 compared with day 28. Baseline and thrombin- or fMLF-induced neutrophil activation and procoagulant status were significantly lower on day 0 compared with day 28. We demonstrate that during the acute phase of COVID-19 compared with the convalescent phase, platelets are more responsive while neutrophils are less responsive. COVID-19 is associated with thromboembolic events where platelet activation and interaction with leukocytes may play an important role.

## Introduction

Coronavirus disease 2019 (COVID-19) has emerged as a pandemic disease caused by the severe acute respiratory syndrome coronavirus 2 (SARS-CoV-2). Most individuals infected with SARS-CoV-2 develop mild respiratory symptoms, however some patients develop a severe form of COVID-19 comprising a requirement for noninvasive ventilation or intensive care unit (ICU) treatment and thromboembolic events. The pathogenesis of this progressive deterioration of lung function has important similarities with the local and systemic inflammation that occurs in Acute Respiratory Distress Syndrome (ARDS)[1]. Early observations report that severe disease was associated with a hyperinflammatory cytokine storm and upregulation of signature cytokines IL-6, TNF-α, and IL-10 [2]. This has been confirmed in subsequent studies and extensive immune mediator profiling has been used to define distinct immune phenotypes of disease [3].

Pathological dysregulation of systemic inflammation contributes to organ damage in sepsis [4] and COVID-19 is emerging as a sepsis-like syndrome. Similar to sepsis, the coagulation system is activated and dysregulated in severe COVID-19, however with distinct patterns of dysregulation [5]. Importantly, thromboembolic complications can occur and antithrombotic therapy may be beneficial [6]. The coagulation system is comprised of a collaborating system of pro- and anti-coagulation plasma proteins, platelets, and endothelial cells, all of which may be activated during inflammation. It is important to fully elucidate the underlying mechanisms contributing to coagulopathy in COVID-19.

Platelets contribute to the dysregulated inflammatory response in ARDS [7] and sepsis [8]. Platelets are important innate immune cells and key mediators of hemostasis and thrombosis [9,10]. During an infection, platelets can recognize pathogens including virus, using Toll-like receptors, complement receptors, and immunoglobulin receptors [11]. Activated platelets adhere and aggregate to the activated endothelium or to one another to form thrombi but also release pro-inflammatory factors that modulate leukocyte function either via released products or direct binding to leukocytes [12]. CD62P upregulation on activated platelets and binding to leukocyte PSGL-1 is critical for platelet–leukocyte complex formation. Platelet activation is also mediated by diverse agonists including thrombin (generated from activation of the coagulation cascade) or ADP (released from activated platelets).

Platelets likely occupy an important crossroads between immune and coagulation dysfunction in COVID-19. Thrombocytopenia has been reported to occur in severe COVID-19 [13,14]. The mechanisms contributing to thrombocytopenia in COVID-19 have not been elucidated and it is important to determine whether this thrombocytopenia is preceded by platelet activation. The aim of this study was to investigate platelet activation in consecutive blood samples from COVID-19 patients throughout the course of disease progression over 28 days.

## Methods

### Study Design

**Figure 1.**
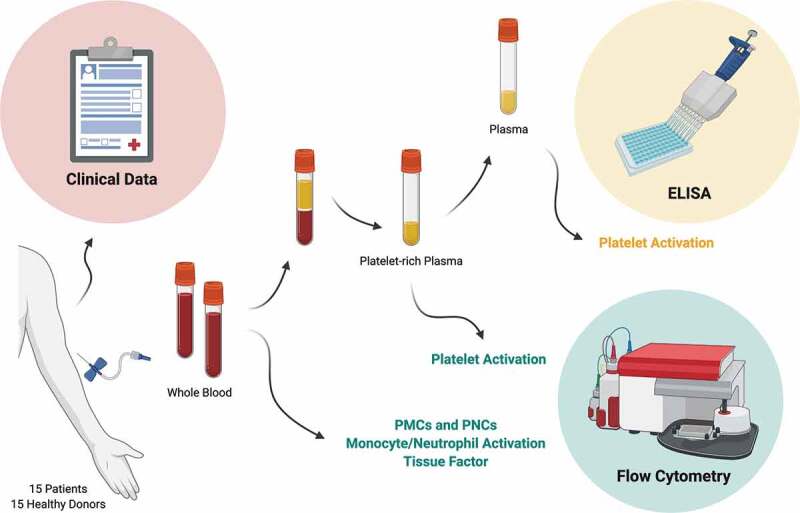
**Schematic diagram of the study protocol**. Clinical data were obtained from patients. Whole blood samples were obtained from patients and healthy donors. Platelet-rich plasma (PRP) was prepared from whole blood. Subsequently, plasma was prepared from PRP. Platelet-monocyte complex (PMC) and platelet-neutrophil complex (PNC) formation, monocyte and neutrophil activation, and upregulation of tissue factor were measured in whole blood by flow cytometry. Platelet activation was measured in PRP and plasma by flow cytometry and ELISA, respectively. This figure was created with BioRender.com

Patients >18 years of age who were admitted to the Clinic of Infectious Diseases, Skåne University Hospital, Lund, between May to July 2020 with COVID-19 (confirmed by RT-PCR) were included in the study after oral and written consent was obtained (*n* = 15). The study protocol is summarized in Figure 1. Consecutive blood samples were obtained during hospitalization on the day of recruitment (day 0) and days 3, 7, 10 and 14. When applicable, blood sampling on day 28 was obtained at a follow-up appointment or during hospitalization. Patient samples were coded at the hospital prior to transporting to the lab. Blood samples from healthy donors were obtained on one occasion after oral and written consent (*n* = 15), and were used to define the reference range of the experimental assays. The study (2020–01747) and the recruitment of healthy donors (2015/801) were approved by the Swedish Ethical Review Authority, and in accordance with the Declaration of Helsinki.

### Blood Collection and Preparation

Whole blood was obtained in BD Vacutainer sodium citrate tubes. Platelet-rich plasma (PRP) was prepared from citrated whole blood by centrifugation at 150 x *g* for 10 min and used directly or fixed with an equal volume of 2% (v/v) formaldehyde in PBS and stored up to three days at 4°C until use. Platelet-poor plasma (referred to as plasma in this study) was prepared from PRP by centrifugation at 2000 x *g* for 10 min and stored at −20°C until use.

### Reagents

Thrombin was purchased from Chrono-log. The anticoagulant peptide H-Gly-Pro-Arg-Pro-NH_2_ (cat. no. H-1998) was purchased from Bachem. Adenosine diphosphate (ADP), formyl-methionyl-leucyl phenylalanine (fMLF), HEPES, and NaCl were purchased from Sigma. KCl, MgS0_4_, and formaldehyde were purchased from Merck. Phosphate-buffered saline (PBS; 0.14 M NaCl, 0.0027 M KCl, 0.0101 M phosphate buffer, pH 7.4; Medicago) was purchased from Saveen & Werner. eBioscience™ 1-step Fix/Lyse Solution (10X) was purchased from Invitrogen, ThermoFisher Scientific. The following fluorescently conjugated antibodies were purchased from BD Pharmingen: CD42a-FITC (clone ALMA.16), CD61-PE (clone VI-PL2), CD11b-PE-Cy™5 (clone ICRF44), and κ isotype control IgG1-PE (clone MOPC-31 C); BD Biosciences: CD62P-PE (clone AC1.2); and eBioscience™, Invitrogen, ThermoFisher Scientific: CD45-FITC (clone HI30), CD142-APC (clone HTF-1), κ isotype control IgG1-FITC (clone P3.6.2.8.1), and κ isotype control IgG1-APC (clone P3.6.2.8.1).

### Flow Cytometry

PRP was diluted 1:5 in HEPES buffer (pH 7.4; 10 mM HEPES, 150 mM NaCl, 5 mM KCl, and 1 mM MgS0_4_) and stimulated with physiological agonists (5 μM ADP or 1 U/ml thrombin) or HEPES buffer (to determine baseline level) for 15 min. Subsequently, samples were incubated with antibodies (1:10) CD42a-FITC (resting and activated platelets) and CD62P-PE (activated platelets) for 15 min, then fixed with 2% (v/v) formaldehyde in PBS for 1 h.

Citrated whole blood was stimulated with physiological agonists (1 U/ml thrombin or 1 μM fMLF) or HEPES buffer for 15 min. Samples were then incubated with antibodies (1:10) CD45-FITC (gating neutrophils and monocytes), CD61-PE (platelet-positive events), CD11b-PE-Cy™5 (leukocyte activation), and CD142-APC (tissue factor) for 15 min. Whole blood samples were fixed with 1X 1-step Fix/Lyse Solution for 1 h, pelleted at 500 x *g* for 5 min, and resuspended in PBS.

Prior to the addition of thrombin, the synthetic anticoagulant peptide H-Gly-Pro-Arg-Pro-NH_2_ was added to PRP or whole blood to prevent fibrin clot formation. Isotype controls were used to determine nonspecific background signals (see **Supplemental Table 1**). All steps were performed at room temperature. Fixed samples were analyzed on a BD Accuri C6 Plus flow cytometer (BD Biosciences). For PRP, logarithmic settings were used, the FSC-H threshold was set at 45,000, and 100,000 events were collected per sample. For whole blood, linear settings were used, the FSC-H threshold was set at 150,000, and 20,000 events were collected per sample. Representative gating strategies and histograms are presented in **Supplemental Figures 1** and **2**. While neutrophils are the most abundant granulocyte, other granulocytes may be included in our neutrophil gate (CD45-leukocytes with high granularity).

### ELISA

ELISA kits were used to measure plasma levels of soluble CD62P (Diaclone) and cytokines IL-6 and IL-10 (Quantikine® ELISA, Bio-Techne, R&D Systems) according to manufacturer’s instructions.

### Statistics

Statistical analyses were performed using GraphPad Prism 9 software. Comparisons were analyzed for statistical significance using Wilcoxon matched-pairs signed rank test or Friedman test with Dunn’s multiple comparisons test. Results were deemed significant for comparisons where *P* < .05.

## Results

### Patient Characteristics

Fifteen patients with confirmed COVID-19 were included in the study. Table I summarizes clinical characteristics and patient data grouped according to the requirement of noninvasive ventilation and/or oxygen treatment. Three patients presented severe forms of COVID-19 and required noninvasive ventilation or ICU treatment. Two of these patients died during the study period prior to blood sampling on day 28. Seven patients had moderate COVID-19, with oxygen support from one to up to six liters per minute. Five patients presented with mild disease and did not require oxygen treatment. Patients 5, 7, and 12 had nosocomial COVID-19.

**Table I.** Clinical characteristics of patients with COVID-19

*Patient*	*Age/Sex*	*Prior to day 0 (d)*		*Platelet count* on day 0* (10^9^/l)	*CCI*	*Oxygen* *treatment*	*NIV/* *Optiflow*	*Care unit*	*Outcome*
		*Onset of* *symptoms*	*Hospital* *admission*						
2	M88	10	5	150	5	Yes	Yes	Intermediate	Deceased (d 29) **
3	F86	10	3	101 (d3)	12	Yes	Yes	Standard	Deceased (d 8) **
7	F74	11	3	325	5	Yes	Yes	Intensive	Survived
1	F72	7	3	349 (adm)	9	Yes	No	Standard	Survived
4	F85	1	27	383	4	Yes	No	Standard	Survived
9	M69	10	3	187	2	Yes	No	Standard	Survived
10	F53	5	1	286	2	Yes	No	Standard	Survived
13	M53	9	2	196 (adm)	1	Yes	No	Standard	Survived
14	F45	12	2	234	0	Yes	No	Standard	Survived
15	M41	9	1	190	0	Yes	No	Standard	Survived
5	M80	8	3	270	7	No	No	Standard	Survived
6	F71	4	10	102 (adm)	9	No	No	Standard	Survived
8	F75	14	2	273 (adm)	3	No	No	Standard	Survived
11	F34	7	2	206	0	No	No	Standard	Survived
12	F59	8	2	234 (adm)	2	No	No	Standard	Survived

*Charlson comorbidity index (CCI)* [15], noninvasive ventilation (NIV), * day of admission (adm) or day 3 (d3), ** in-hospital mortality

The median age in the study cohort was 71 years (interquartile range, IQR, 53–80 years) and the majority were female (67%). Prior to sampling on day 0, the median days from onset of symptoms was 9 (IQR 7–10 days) and the median days from hospital admission was 3 (IQR 2–3 days). The circulating platelet and WBC counts were within the normal range for all patients where data were available, except in Patient 6 where relative leukopenia was observed (data not shown). Platelet counts are reported from day 0 where data were available, or on the day of admission or study day 3 where indicated (Table I). The relative platelet count in platelet-rich plasma was determined by CD42a-positive events by flow cytometry, which correlated well with clinical platelet counts from day 0 (Spearman r = 0.7833, *P* = .0172) and are reported in **Supplemental Table 2**. Medication prior to admission and anticoagulant treatments are reported in **Supplemental Table 3**. No patients received low molecular weight heparin (LMWH) or direct oral anticoagulant drug (DOAC) treatments from day 14 to day 28 except when stated under prior medications before admission.

In this study, we investigate responses during the acute phase of COVID-19 (day 0) using paired comparison to the convalescent phase (day 28) within the patient cohort. Healthy controls were used to define the reference range of the experimental assays. Due to local restrictions, individuals of age 70+ were self-isolating and could not be recruited and, therefore, healthy donors have a lower median age of 44 years (IQR 31–60 years). As the majority of patients were discharged within one week of hospitalization, data of all assays performed in the cohort are summarized only from day 0 and day 28. For patients that were sampled up until day 7, the additional data are provided in **Supplemental Figure 3**. For the two patients that died during the study period, only data from day 0 was included for analysis.

### Cytokine Mobilization in Plasma

**Figure 2.**
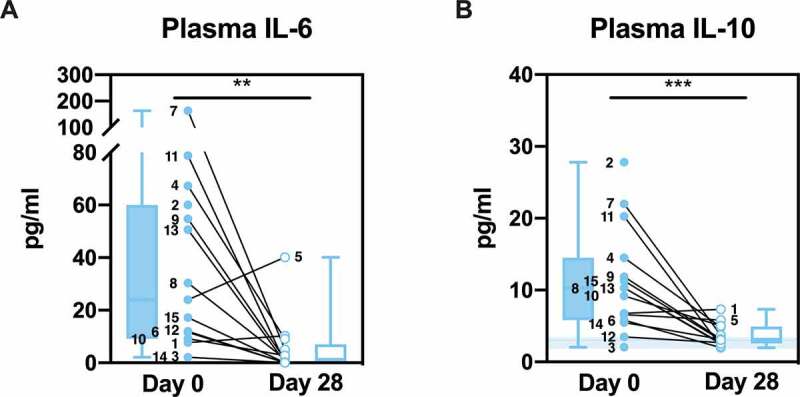
**Cytokine levels in plasma**. Plasma IL-6 (**a**) and IL-10 (**b**) of patients were detected by ELISA and are shown as individual values and box and whisker plots. Healthy controls are shown in the background as the median (*line*) ± interquartile range (*fill*). Statistical analysis was performed using Wilcoxon matched-pairs signed rank test; ** *P* < .01, *** *P* < .001

The plasma levels of the pro-inflammatory cytokine IL-6 and the anti-inflammatory cytokine IL-10 were detected using ELISA. Both cytokines were significantly elevated on day 0 as compared with day 28 (*P* = .0061, IL-6; *P* = .0005, IL-10), confirming cytokine mobilization in the patient cohort (Figure 2). Patient 7 exhibited the highest IL-6 and the second highest IL-10 levels and was the only patient in the cohort that required ICU supportive care.

### Platelet Activation in Platelet-rich Plasma

**Figure 3.**
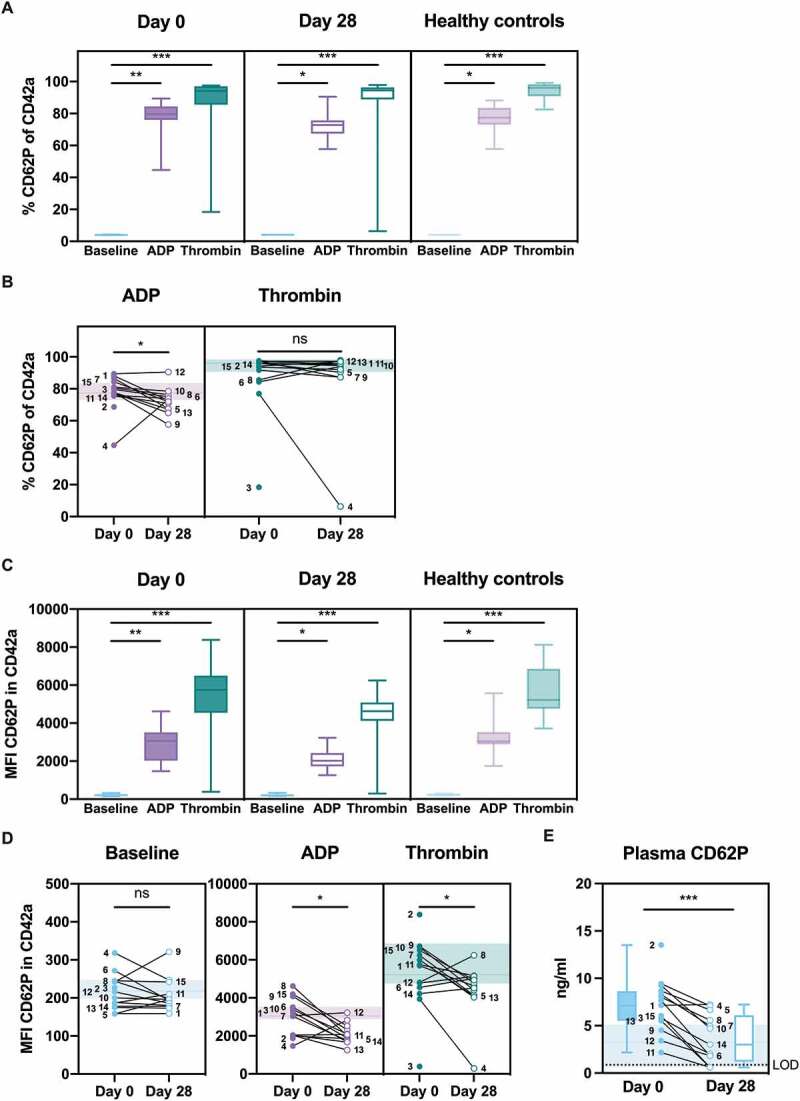
**Platelet activation signatures in plasma**. Platelet-rich plasma was stimulated with HEPES (to determine baseline levels), 5 μM ADP, or 1 U/ml thrombin (**a-d**). Platelet activation marker CD62P was determined as the percentage (**a** and **b**) or the median fluorescence intensity (MFI; **c** and **d**) of CD42a-positive platelets by flow cytometry. The baseline percentage of platelet activation was approximately 4% (**a**). (**e**) Soluble CD62P in non-stimulated plasma was determined by ELISA and is shown as individual values and box and whisker plots. The limit of detection (LOD) is shown as a dotted line. Healthy controls are shown in the background as the median (*line*) ± interquartile range (*fill*) (**b, d**, and **e**). Statistical analysis was performed using Friedman test with Dunn’s multiple comparisons test (**a** and **c**) or Wilcoxon matched-pairs signed rank test (**b, d**, and **e**); ns = not significant, * *P* < .05, ** *P* < .01, *** *P* < .001

Upregulation of CD62P to the activated platelet surface was determined using flow cytometry of platelet-rich plasma (PRP) in the presence or absence of *ex vivo* stimulation with platelet agonists, ADP or thrombin. Platelets from the majority of patients responded to *ex vivo* stimulation on day 0 and also on day 28, both in terms of percent positive platelets in the population (day 0: *P* = .007, ADP; *P* < .001, thrombin; day 28: *P* = .01, ADP; *P* < .001, thrombin; Figure 3A) and the median fluorescence intensity (MFI) of CD62P per cell (day 0: *P* = .007, ADP; *P* < .001, thrombin; day 28: *P* = .01, ADP; *P* < .001, thrombin; Figure 3C). Patient 3 failed to respond to thrombin on day 0 and Patient 4 failed to respond to thrombin on day 28. In both cases, platelets became activated in response to ADP, demonstrating that the platelet population can respond to activation by agonists other than thrombin. Healthy controls also responded significantly to both agonists (*P* = .02, ADP; *P* < .001, thrombin).

Comparison of the same data but focusing on changes in platelet activation between day 0 and day 28 in terms of individual agonists showed no significant increase in baseline activation between the two time points, both in terms of percent positive platelets (approximately 4%, data not shown) and the MFI of CD62P per cell (Figure 3D). The percent positive platelets upon addition of ADP and the MFI of CD62P per cell upon addition of either ADP (*P* = .02) or thrombin (*P* = .02) were significantly higher on day 0 as compared with day 28 (Figure 3D). While addition of thrombin did not differ significantly in terms of percent positive platelets, the fold increase (day 0 relative to day 28) correlated significantly with the fold increase of the MFI of CD62P (Spearman r = 0.8571, 95% confidence interval 0.5679–0.9580, *P* = .0004). The data suggest that, for the majority of patients, the platelet population was more responsive to stimulation on day 0 as compared with day 28.

Following activation, CD62P is released from the platelet surface. Platelet activation was, therefore, further assessed using ELISA to determine soluble CD62P levels in unstimulated plasma samples. Baseline CD62P levels were significantly higher on day 0 as compared with day 28 (*P* = .0005; Figure 3E). This confirms that platelet activation had occurred in the majority of patients.

### Platelet-leukocyte Complex Formation in Blood

**Figure 4.**
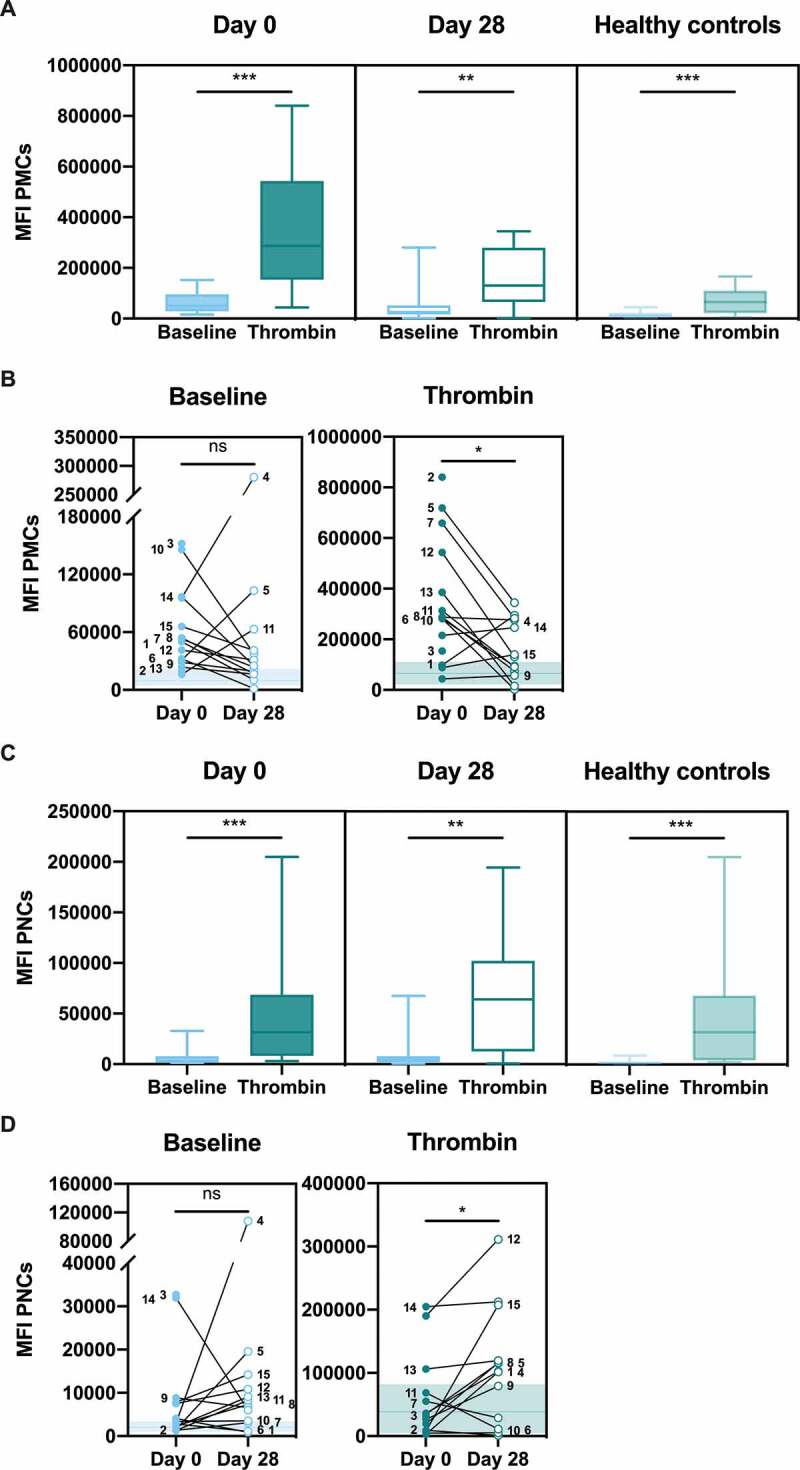
**Platelet activation signatures in whole blood; platelet-monocyte complex (PMC) and platelet-neutrophil (PNC) formation**. Citrated whole blood was stimulated with HEPES (to determine baseline levels) or 1 U/ml thrombin. The median fluorescence intensity (MFI) of CD61 (platelet-positive events) on CD45-positive monocytes (**a** and **b**) or neutrophils (**c** and **d**) were determined by flow cytometry. Healthy controls are shown in the background as the median (*line*) ± interquartile range (*fill*) (**b** and **d**). Statistical analysis was performed using Wilcoxon matched-pairs signed rank test; ns = not significant, * *P* < .05, ** *P* < .01, *** *P* < .001

Following activation, platelets can bind to circulating leukocytes. Platelet-monocyte complex (PMC) or platelet-neutrophil complex (PNC) formation was determined using flow cytometry of whole blood in the presence or absence of *ex vivo* stimulation with the platelet agonist thrombin. Platelet-positive events were determined by staining with CD61. There was a significant increase in PMC formation (Figure 4A) and PNC formation (Figure 4C) in response to thrombin stimulation in patients on day 0 (*P* < .001, PMCs; *P* = .0003, PNCs) and day 28 (*P* = .002, PMCs; *P* = .0017, PNCs) and in healthy controls (*P* < .001, PMCs; *P* = .0001, PNCs). As was the case for platelet activation in PRP, Patient 4 failed to generate PMCs or PNCs in response to thrombin stimulation on day 28, and this is likely explained by the observation that this patient had highly elevated PMC and PNC formation already at baseline (without stimulation) on day 28.

Comparison of the same data but focusing on changes in PMC and PNC formation between day 0 and day 28 showed no significant increase in baseline activation between the two timepoints, however, there was a tendency toward increased PMC formation on day 0 as compared with day 28 (Figures 4B and 4D). PMC formation upon addition of thrombin was significantly higher (*P* = .03) on day 0 as compared with day 28 (Figure 4B). PNC formation upon addition of thrombin was significantly lower on day 0 as compared with day 28 (*P* = .03), however, more heterogenous changes were observed in this neutrophil population (Figure 4D). The results suggest that, in the majority of patients, PMC formation was more responsive to thrombin stimulation on day 0, while PNC formation was less responsive to thrombin stimulation on day 0 as compared with day 28.

### Monocyte Activation and Procoagulant Status in Blood

**Figure 5.**
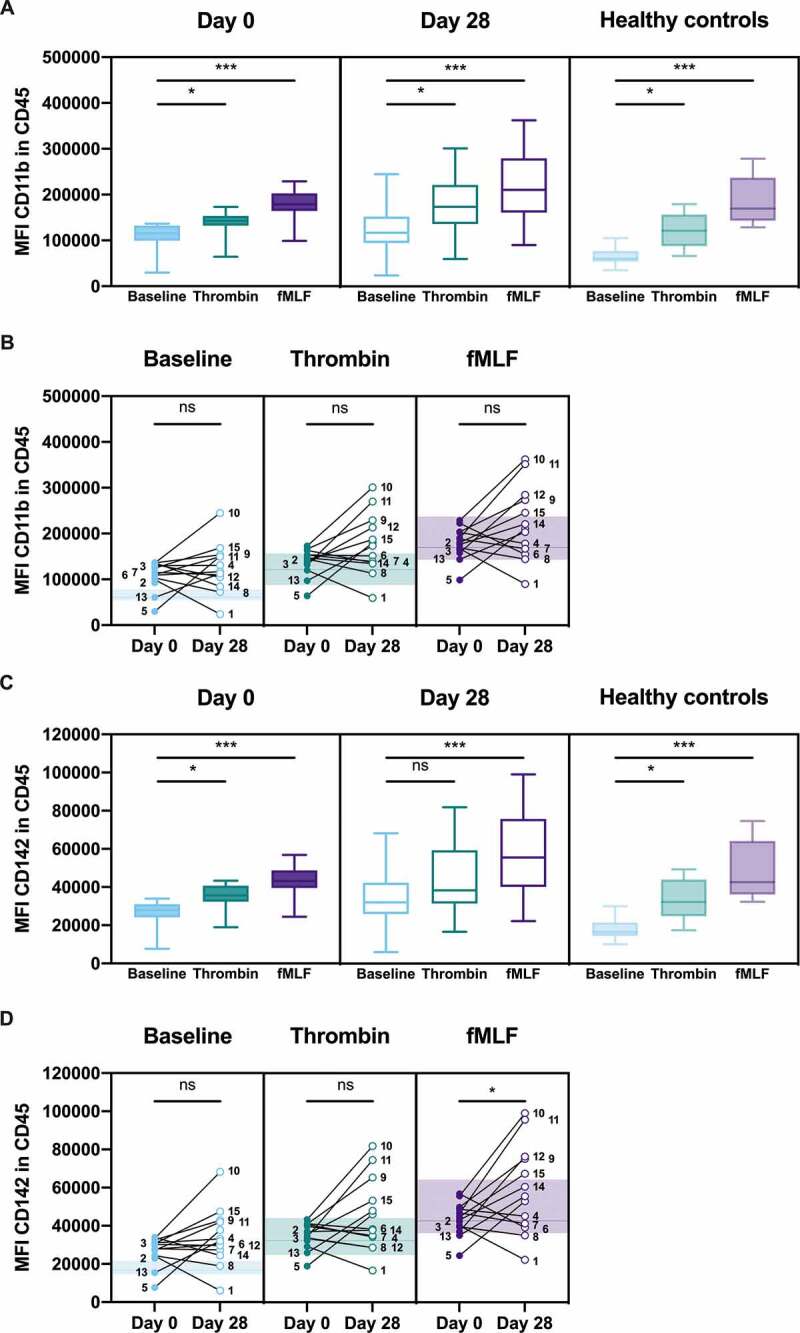
**CD11b and tissue factor upregulation on monocytes in whole blood**. Citrated whole blood was stimulated with HEPES (to determine baseline levels), 1 U/ml thrombin, or 1 μM fMLF. The median fluorescence intensity (MFI) of CD11b (activation marker) or CD142 (tissue factor) on CD45-positive monocytes were determined by flow cytometry. Healthy controls are shown in the background as the median (*line*) ± interquartile range (*fill*) (**b** and **d**). Statistical analysis was performed using Friedman test with Dunn’s multiple comparisons test (**a** and **c**) or Wilcoxon matched-pairs signed rank test (**b**, and **d**); ns = not significant, * *P* < .05, *** *P* < .001

Flow cytometry was used to determine upregulation of CD11b (activation marker) or CD142 (tissue factor) to the monocyte surface in the presence or absence of *ex vivo* stimulation with the monocyte agonist, fMLF, or the platelet agonist, thrombin, in whole blood. There was a significant increase in the MFI of CD11b (Figure 5A) in response to thrombin and fMLF stimulation on day 0 (*P* = .01, thrombin; *P* < .001, fMLF) and on day 28 (*P* = .02, thrombin; *P* < .001, fMLF). The MFI of CD142 was significantly increased by fMLF on both day 0 (*P* < .001) and day 28 (*P* < .001), however thrombin stimulation failed to significantly increase CD142 on day 28 (Figure 5C). Significant increases in the MFI of CD11b and CD142 were observed in healthy controls (*P* = .02, thrombin; *P* < .001, fMLF). As expected, monocyte activation was more pronounced in response to leukocyte agonist fMLF as compared to the platelet agonist thrombin.

Comparison of the same data but focusing on changes in activation between day 0 and day 28 showed no significant difference in CD11b or CD142 upregulation at baseline or upon stimulation with thrombin. For fMLF stimulation, there was no significant difference in CD11b upregulation (Figure 5B), but significantly higher CD142 on day 28 than day 0 was observed (*P* = .05; Figure 5D). The results for monocyte activation were highly heterogenous within the cohort, with two apparent subpopulations. In one subpopulation, there was a trend of increased CD11b and CD142 on day 28 as compared with day 0, while the second subpopulation showed decreased or unchanged CD11b and CD142 on day 28 as compared with day 0 (Figures 5B and 5D).

### Neutrophil Activation and Procoagulant Status in Blood

**Figure 6.**
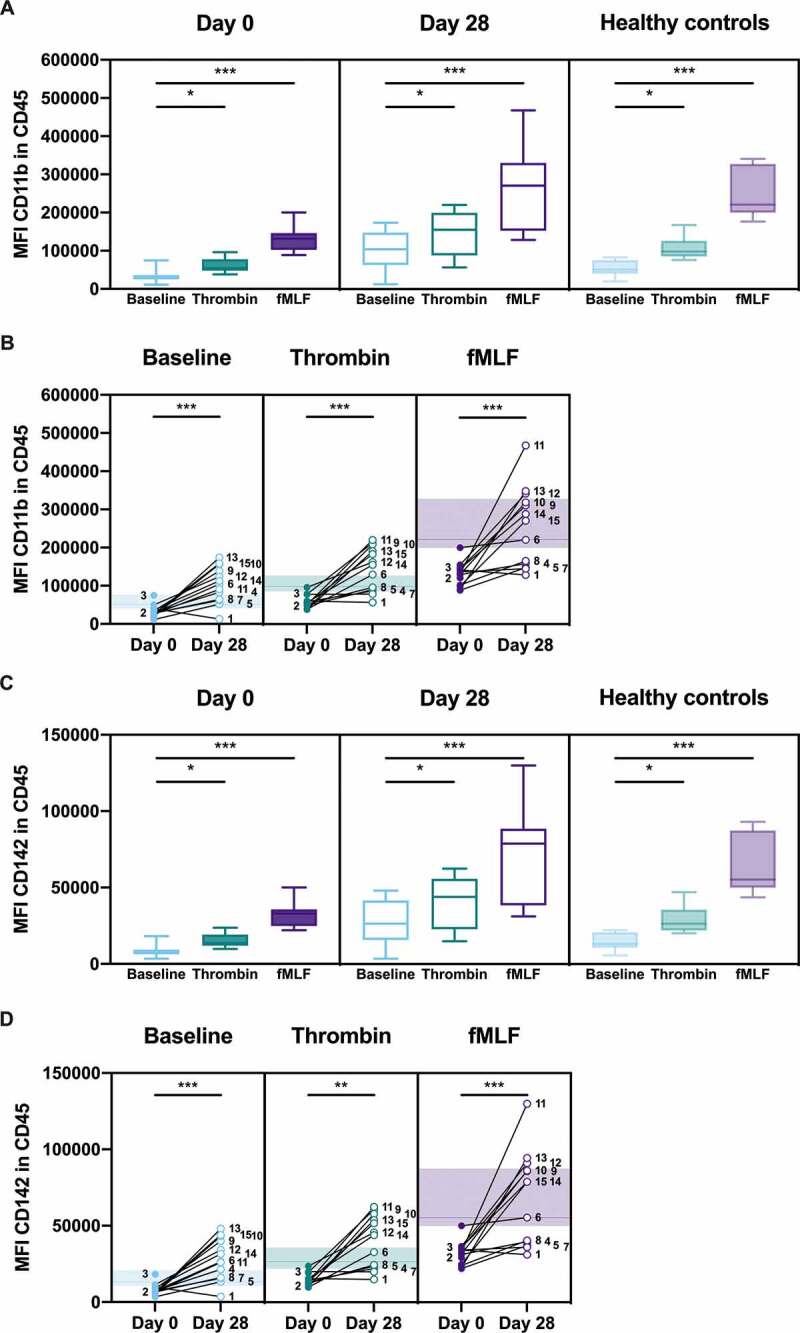
**CD11b and tissue factor upregulation on neutrophils in whole blood**. Citrated whole blood was stimulated with HEPES (to determine baseline levels), 1 U/ml thrombin, or 1 μM fMLF. The median fluorescence intensity (MFI) of CD11b (activation marker) or CD142 (tissue factor) on CD45-positive neutrophils were determined by flow cytometry. Healthy controls are shown in the background as the median (*line*) ± interquartile range (*fill*) (**b** and **d**). Statistical analysis was performed using Friedman test with Dunn’s multiple comparisons test (**a** and **c**) or Wilcoxon matched-pairs signed rank test (**b**, and **d**); * *P* < .05, ** *P* < .01, *** *P* < .001

Flow cytometry was used to determine upregulation of CD11b or tissue factor to the neutrophil surface in the presence or absence of *ex vivo* stimulation with the neutrophil agonist, fMLF, or the platelet agonist, thrombin, in whole blood. There was a significant increase in the MFI of CD11b (Figure 6A) and CD142 (Figure 6C) in response to thrombin and fMLF stimulation on day 0 (*P* = .01, thrombin; *P* < .001, fMLF) and on day 28 (*P* = .02, thrombin; *P* < .001, fMLF). Significant increases in the MFI of CD11b and CD142 were also observed in healthy controls (*P* = .02, thrombin; *P* < .001, fMLF). As expected, neutrophil activation was more pronounced in response to leukocyte agonist fMLF as compared to the platelet agonist thrombin.

Comparison of the same data but focusing on changes in activation between day 0 and day 28 showed that neutrophil activation was significantly decreased on day 0 as compared with day 28 under all conditions; baseline (*P* < .001, CD11b; *P* < .001, CD142), thrombin stimulation (*P* < .001, CD11b; *P* = .001, CD142), or fMLF stimulation (*P* < .001, CD11b; *P* < .001, CD142) (Figures 6B and 6D). Furthermore, neutrophil activation was lower than the range in healthy controls for the majority of patients on day 0 under all conditions; baseline, upon thrombin stimulation, and upon fMLF stimulation (Figures 6B and 6D). The results within the cohort were heterogenous, whereby seven patients exhibited an obvious increase on day 28, while 6 patients exhibited a relatively small increase or no change. Collectively, the results demonstrate that circulating neutrophils were less responsive in patients on day 0 compared with day 28, indicating that activation may already have occurred.

## Discussion

Complementary assays were used to determine the activation status of the circulating platelet pool and modulation of neutrophil and monocyte activation and procoagulant status over time from day 0 to day 28 post-COVID-19 diagnosis. The small cohort size and low incidence of severe disease is an important limitation of our study, however significant changes were observed during the course of disease. The majority of patients in the cohort were mildly or moderately ill, however a clear cytokine mobilization occurred during the acute phase of infection. Patient 2 exhibited the highest levels of IL-10 on day 0, but day 28 was not obtained as the patient died prior to blood sampling. Additionally, Patient 3 showed elevated levels of IL-6 and IL-10 but follow-up blood sampling was not obtained as the patient died shortly after admission to the hospital.

An important strength of our study is the investigation of platelet activation and leukocyte activation in consecutive samples from a patient cohort both during acute phase COVID-19 and the convalescent phase using paired analysis. Our observation that platelet activation occurs in acute COVID-19 is supported in the recent literature. Platelets from COVID-19 patients exhibit altered gene expression, increased activation and formation of platelet-leukocyte complexes, and increased release of platelet-derived microparticles [16–19]. Patient cohorts with predominately severe COVID-19 have been reported to have significantly elevated baseline CD62P (P-selectin) surface expression compared to healthy donors [16,17]. In contrast, in the present patient cohort consisting of primarily mild and moderate COVID-19, baseline CD62P surface expression levels were similar between the acute phase and convalescent phase. This agrees well with the finding of similar levels of CD62P between patients with asymptomatic or mild COVID-19 and healthy donors [17]. In our study, platelet populations were significantly more responsive to classical platelet agonists, ADP and thrombin, during the acute phase as compared with the convalescent phase. This is in agreement with a report of significantly elevated ADP-induced surface P-selectin in patients with COVID-19 compared to healthy donors [16]. A consistent observation among all studies, including the present study, is elevated plasma levels of platelet-derived proteins in patients with COVID-19. Soluble CD62P in plasma was significantly increased during the acute phase, indicating that activation had already occurred *in vivo*. This is in contrast to our observations of similar baseline levels of surface CD62P expression on platelets. It is important to note that, due to platelet turnover, the soluble CD62P may not necessarily be derived from the same platelet population that was analyzed for surface CD62P by flow cytometry. PMCs in our patient cohort were higher at baseline in the majority of patients, albeit not significant, during the acute phase compared with the convalescent phase while thrombin-induced PMCs were significantly higher. We propose that studies of platelet activation should utilize multiparameter assessments of platelet activation at both baseline and post *ex vivo* stimulation with weak and strong agonists in order to account for potential technical limitations of single assays.

Collectively, we demonstrate that platelet activation is an early response to mild or moderate COVID-19 and is not only associated with severe disease. Importantly, platelet-rich thrombi have been observed in multiple organs on autopsy post COVID-19, implying that platelet activation and aggregation may contribute to organ damage [20].

We report that distinct leukocyte phenotypes were evident during the acute phase of COVID-19, although this was not the main focus of the study and a limited number of leukocyte activation markers were investigated. Monocytes are an important source of procoagulant tissue factor and cytokines and monocyte dysregulation has been reported in COVID-19 [3,21]. Monocyte activation was highly heterogeneous with a tendency toward increased responsiveness on day 0 while neutrophil activation was more homogenous within the patient cohort. The neutrophil population was less responsive on day 0 compared with day 28, and response to stimulation on day 0 was lower than the range in healthy controls, which implies that *in vivo* activation may have already occurred. This may reflect a circulating pool of degranulated neutrophils. Platelets contribute to the recruitment of neutrophils to the local site of inflammation in ARDS and contribute to tissue injury through inflammatory damage and thrombus formation [7]. This has also been reported to occur in COVID-19 where neutrophil extracellular trap formation occurs in the lungs of severely ill patients [22]. Biomarker profiling of plasma from COVID-19 patients has demonstrated a significant neutrophil activation profile [23]. In agreement with our findings, a recent flow cytometry-based profile of blood-derived neutrophils also reported a downregulation of neutrophil CD11b during acute disease [24].

As platelets have both a coagulation and an immune role, platelet biomarkers have the potential to reveal therapeutic targets relevant for either inflammation or coagulation dysfunction in COVID-19. The platelet activation phenotype observed in COVID-19 further demonstrates that platelets can respond to virus infection. Platelet activation and microparticle release occurs in response to the respiratory influenza A virus (IAV) [25] and occurs in the circulation of patients infected with the pandemic H1N1 IAV [26]. IAV particles can be taken up by platelets, resulting in platelet granule release and platelet-dependent enhancement of neutrophil extracellular trap formation [27]. In a mouse model of IAV infection, platelet aggregation promoted lung damage and antiplatelet therapy protected mice from lethal infection [28]. It is possible that platelets also take up the SARS-CoV-2 virus as a recent report has demonstrated that hyperactivated platelets from COVID-19 patients express SARS-CoV-2 RNA [18]. While monitoring platelet counts is proposed to be valuable in COVID-19 [29], monitoring platelet activation may provide better predictive value since it may precede changes in platelet counts. Interestingly, a recent comprehensive proteomic analysis of biomarkers in the plasma of COVID-19 patients identified biomarkers of platelet degranulation as robust candidates [30]. Antiplatelet therapy has been suggested for ARDS and sepsis and may well be beneficial in COVID-19. Protective or therapeutic effects of antiplatelet therapy have theoretical benefits during COVID-19 progression [31] and warrants further investigation.

In conclusion, in the cohort of mildly and moderately ill COVID-19 patients, we demonstrate that circulating platelets are more responsive and platelet activation occurs during the acute phase compared with the convalescent phase. Monocytes are heterogeneous in their responsiveness but circulating neutrophils are significantly less responsive during the acute phase of infection. Understanding platelet activation and interaction with leukocytes during disease progression may reveal new strategies to regulate host inflammation in COVID-19.
